# Optimizing human α-galactosidase for treatment of Fabry disease

**DOI:** 10.1038/s41598-023-31777-4

**Published:** 2023-03-23

**Authors:** William C. Hallows, Kristen Skvorak, Nick Agard, Nikki Kruse, Xiyun Zhang, Yu Zhu, Rachel C. Botham, Chinping Chng, Charu Shukla, Jessica Lao, Mathew Miller, Antoinette Sero, Judy Viduya, Moulay Hicham Alaoui Ismaili, Kerryn McCluskie, Raphael Schiffmann, Adam P. Silverman, Jin-Song Shen, Gjalt W. Huisman

**Affiliations:** 1 Codexis Inc.,, 200 Penobscot Drive, Redwood City, CA 94063 USA; 2 Institute of Metabolic Disease, Baylor Research Institute, Dallas, TX 75246 USA; 3 Present Address: Genentech, South San Francisco, CA 94080 USA; 4 Present Address: Fornia BioSolutions Inc US, Hayward, CA 94545 USA; 5 Present Address: Octant, Emeryville, CA 94608 USA; 6 Present Address: Glycomine, San Mateo, CA 94070 USA; 7 Present Address: 4D Molecular Therapeutics, Emeryville, CA 94608 USA

**Keywords:** Biomedical engineering, Biochemistry, Molecular medicine

## Abstract

Fabry disease is caused by a deficiency of α-galactosidase A (GLA) leading to the lysosomal accumulation of globotriaosylceramide (Gb3) and other glycosphingolipids. Fabry patients experience significant damage to the heart, kidney, and blood vessels that can be fatal. Here we apply directed evolution to generate more stable GLA variants as potential next generation treatments for Fabry disease. GLAv05 and GLAv09 were identified after screening more than 12,000 GLA variants through 8 rounds of directed evolution. Both GLAv05 and GLAv09 exhibit increased stability at both lysosomal and blood pH, stability to serum, and elevated enzyme activity in treated Fabry fibroblasts (19-fold) and GLA^–/–^ podocytes (10-fold). GLAv05 and GLAv09 show improved pharmacokinetics in mouse and non-human primates. In a Fabry mouse model, the optimized variants showed prolonged half-lives in serum and relevant tissues, and a decrease of accumulated Gb3 in heart and kidney. To explore the possibility of diminishing the immunogenic potential of rhGLA, amino acid residues in sequences predicted to bind MHC II were targeted in late rounds of GLAv09 directed evolution. An MHC II-associated peptide proteomics assay confirmed a reduction in displayed peptides for GLAv09. Collectively, our findings highlight the promise of using directed evolution to generate enzyme variants for more effective treatment of lysosomal storage diseases.

## Introduction

Fabry disease is an X-linked inborn error of glycosphingolipid metabolism, characterized by progressive systemic accumulation of glycosphingolipids including globotriaosylceramide (Gb3, GL-3, ceramide trihexoside). The disease is caused by mutations in the α-galactosidase A (GLA) gene that cause a deficiency of the active enzyme in the lysosome^1^. This genetic defect can lead to progressive cardiac disease, renal failure, small fiber peripheral neuropathy, cerebrovascular disease, as well as symptoms that affect other systems^2,3^. The estimated incidence of Fabry disease is 1 in 50,000 live births, though recent newborn screening studies suggest a much higher occurrence in certain populations^4^.

The most widely used treatment for Fabry disease is enzyme replacement therapy (ERT) with recombinant human GLA (rhGLA). Two preparations, agalsidase alfa (Replagal^®^, Takeda) and agalsidase beta (Fabrazyme^®^, Sanofi), have been shown to slow disease progression when administered regularly and have been approved for the treatment of Fabry disease. Other GLA ERTs that have been recently approved or are in late-stage development include pegunigalsidase alfa^5^, a plant cell culture-expressed, chemically-modified version of GLA, and moss-cell produced GLA^6^. A small molecule therapy, migalastat (Galafold^®^, Amicus Therapeutics) is an oral chaperone therapy that increases residual enzymatic activity in a subset of patients who have an amenable GLA mutation (30–50% of patients). Other therapies such as glucosylceramide synthase inhibitors (Lucerastat)^7^, and gene^8^ and cell therapies^9^ are currently in clinical development, while additional therapeutic options are also being investigated^10^.

During ERT, rhGLA is rapidly cleared from serum, requiring patients to receive biweekly infusions that entail significant impacts to the patient’s quality of life and the health care system^11,12^. Even with frequent administration of GLA, patients often continue to exhibit severe disease phenotypes and suffer early mortality^13–15^. The failure of ERT to entirely correct the cause of disease is possibly due to incomplete restoration of enzyme activity in the lysosomes of cells in the key affected tissues of the heart and kidney. This may be due to the instability of rhGLA in blood, leading to most of the active enzyme failing to reach target organs. A second cause of poor delivery of GLA to the target tissues is the development of an immune reaction to the GLA protein. In male patients, infusion reactions and neutralizing anti-drug antibodies (ADAs) are present in up to 73% of patients treated with agalsidase beta and 24% of patients treated with agalsidase alfa^16^. Patients who develop neutralizing ADAs experience attenuation of ERT efficacy over time^16–19^. Suboptimal exposure due to instability, infusion-associated side reactions, and the long-term inhibiting effects of neutralizing antibodies represent major shortcomings for GLA ERT to provide adequate, life-long, efficacious treatment of Fabry disease^20,21^.

The goal of this study was to identify variants of GLA that show improved performance as potential next-generation therapies for Fabry patients. For this purpose we applied directed evolution^22^ to GLA through cycles of semi-rational design of protein libraries, high throughput in vitro activity assays, next-generation sequencing, and bioinformatics, to discover engineered GLA variants with improved properties.

## Results

### Directed evolution of GLA

**Figure 1:**
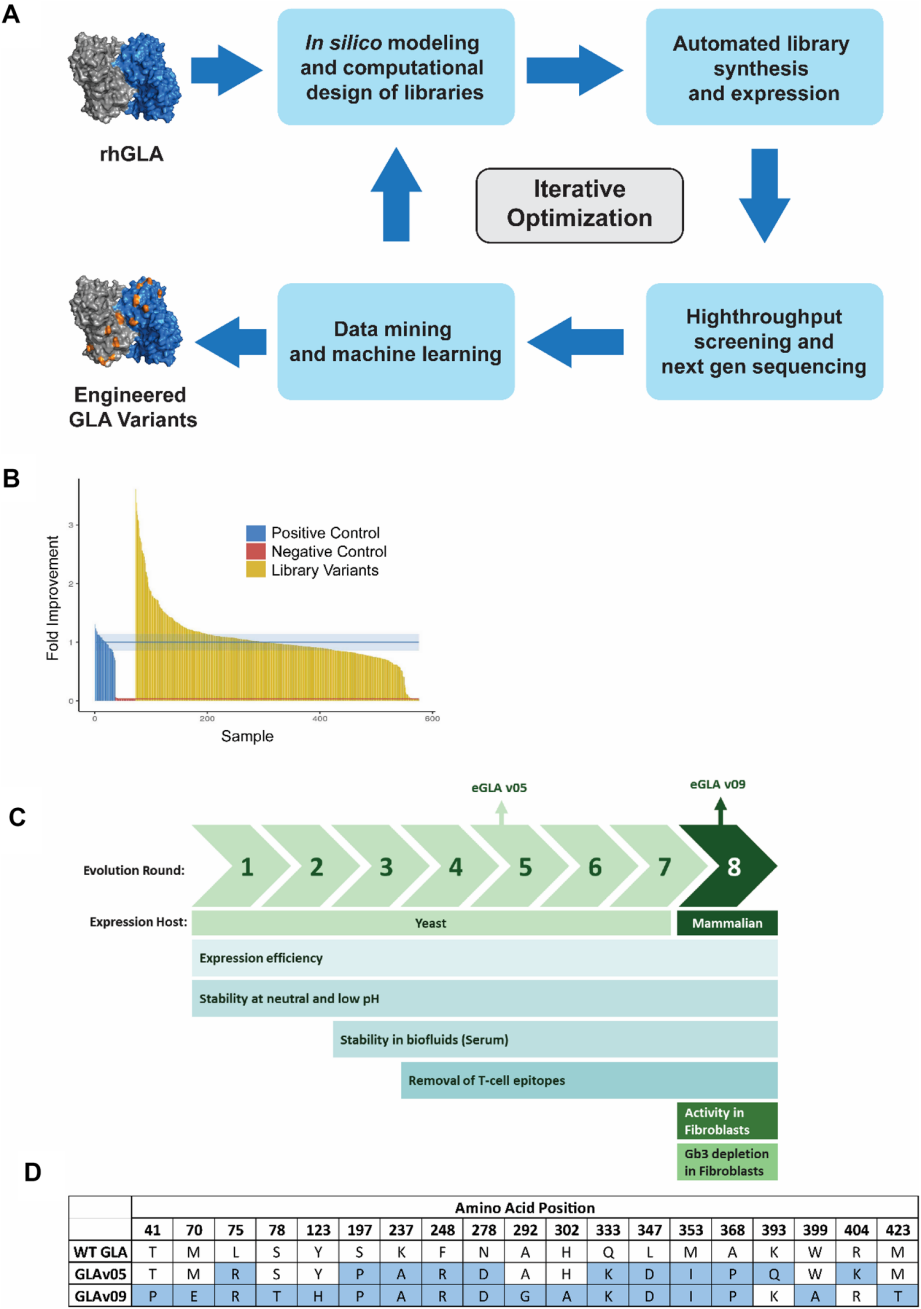
Directed evolution approach for optimizing GLA. (**A**) Enzyme evolution scheme. The published structure of GLA was used to identify positions for mutagenesis in site saturation and combinatorial libraries. Libraries were generated and variants were spatially separated in 96-well plates for screening and DNA sequencing. Beneficial mutations were identified, and the directed evolution cycles were repeated. (**B**) Example of the activity distribution for the Rd 1 GLA variants exposed to pH 7 for 1 h prior to activity measurement. (**C**) Evolution targets and screens over the course of optimization. (**D**) Amino acid changes in GLAv05 and GLAv09 compared to the GLA sequence (Uniprot sequence P06280).

We performed iterative rounds of GLA library generation, high throughput screening, and next generation sequencing (NGS), combined with in silico analyses to identify GLA variants with increased stability in vitro that we hypothesized would lead to improved pharmacological properties. An overview of our approach is shown in Fig. 1.

We designed initial libraries to target key surface residues based on available structural information^23^ while avoiding active site residues to minimize the risk of inadvertently altering substrate specificity. Initially, we generated GLA mutant libraries via both site saturation mutagenesis and multi-site mutagenesis; multi-site libraries were based on amino acid sequences of GLA homologs. The libraries were expressed and secreted from *S. cerevisiae*.

Given that lysosomal ERTs are exposed to neutral pH (pH 7.4) during manufacturing and within the bloodstream after infusion, and then to the acidic pH of the lysosome (pH 4.4) upon uptake in target cells, our first objective was to improve stability across a broad pH range. GLA variants from the various libraries were secreted from *S. cerevisiae* and after incubation at pH 7.4 or pH 4.2, residual GLA enzyme activity was determined using a standard fluorescence assay with 4-methylumbelliferyl α-d-galactopyranoside(4-MU-Gal)^24^. In the first screening round, among the hundreds of variants tested we identified a lysine-to-alanine mutation at position 237 (K237A) that improved stability relative to rhGLA, by 1.5-fold at pH 7 and 2.5-fold at pH 4.2. An example of screening data showing a library tested for pH 7 stability is presented in Fig. 1B.

The next iteration of enzyme optimization was based on the K237A mutant as the parental sequence, and incorporated combinations of other beneficial mutations that had been identified during the first round of evolution. We screened as described above and identified variants with further improvements in stability, the best of which was selected as the parent for the next set of libraries. This cycle was repeated for a total of 8 rounds (7 rounds involving expression in *S. cerevisiae* and a final round incorporating variant expression in HEK293T cells), incorporating mutations from both site-saturation mutagenesis and homologous sequences.

In later rounds of library generation and screening, we adapted screens for additional stability to elevated temperatures, human serum, or in liver lysosomal extracts (Fig. 1C).

In the later rounds, we also sought to remove sequences potentially responsible for GLA immunogenicity by eliminating sequences predicted to bind to major histocompatibility complex class II (MHC-II). We employed the in silico tool from the Immune Epitope Database (IEDB)^25^ to identify sequences with high scores for binding to MHC-II, and we designed libraries that incorporated mutations specifically designed to eliminate such predicted T-cell epitopes.

We selected two variants for further analysis: GLAv05 (which contains 11 mutations) after five rounds of directed evolution in *S. cerevisiae* and GLAv09 (which contains 17 mutations) following the final round of evolution in HEK293T cells (Fig. 1D).

### In vitro characterization of GLA variants

We expressed GLA, GLAv05, and GLAv09 in Expi293 cells and purified them in a two-step process using concanavalin A affinity and size-exclusion chromatography (SEC). While GLA purified as a mixture of monomer and homodimer, GLAv05 and GLAv09 purified as > 95% homodimers directly off the affinity column (Figure S1). We determined the Michalis-Menten kinetic parameters V_max_ and K_m_ by measuring the activity of GLA, GLAv05 and GLAv09 on 4-MU-Gal at pH 4.4 over a range of substrate concentrations (Figure S2). The kinetic parameters for GLAv05 and GLAv09 were similar to those measured for GLA, indicating that enzyme optimization for stability did not significantly alter the catalytic properties of these variants (Table S1).

**Figure 2:**
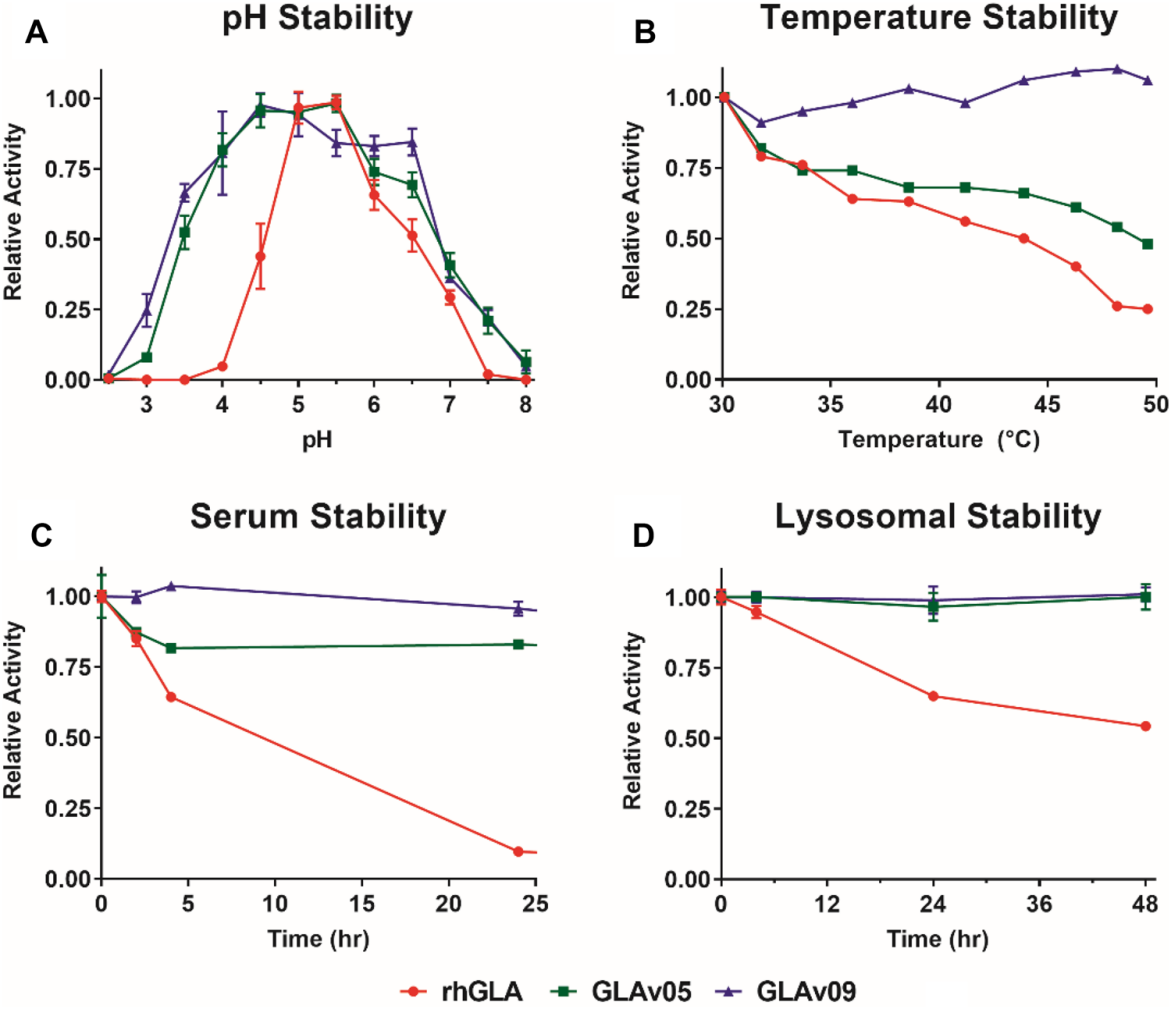
Stability of GLA variants. The activity of each variant (GLA (red circles), GLAv05 (green squares), and GLAv09 (blue triangles) was measured in a 4-MU-Gal hydrolysis assay following the indicated challenge conditions (three technical replicates per sample, except for heat challenge which shows results of a single test at each data point). (**A**) Enzymes were pre-incubated at the indicated pH for 24 h (n = 3). (**B**) Enzymes were pre-incubated at the indicated temperature for 1 h (n = 1). (**C**) Enzymes were incubated in human serum at 37 °C and assayed at indicated timepoints (n = 3). (**D**) Enzymes were incubated in human liver lysosome extracts at 37 °C and assayed at indicated timepoints (n = 3).

We next characterized purified GLA, GLAv05 and GLAv09 for properties that could impact their developability and in vivo efficacy. We incubated the enzymes at pHs ranging from 2 to 8 for 24 h, and then measured their residual activity. The pH stability profiles of GLAv05 and GLAv09 were broader than that of GLA, with the enzymes retaining > 75% of their activity following challenges at pH 4–6.5 (Fig. 2A). GLAv09 showed substantial improvement in thermostability, retaining nearly all activity following a 1-h incubation at 50 °C, whereas both GLA and GLAv05 had limited stability at temperatures > 30 °C (Fig. 2B). We next used differential scanning fluorometry to determine the melting temperatures (T_M_) of GLA, GLAv05, and GLAv09. We compared these values to the T_M_ of GLA with 50 mM 1-deoxygalactonojirimycin (DGJ), a small molecule chaperone that stabilizes the enzyme and increases its T_M_ at both neutral and acidic pH^26^. Both GLAv05 and GLAv09 variants showed increases in T_M_ at pH 4.5 and 7.4 that are similar to the stabilizing effect of DGJ on GLA (Table 1). Finally, we demonstrated that GLAv05 and GLAv09 were stabilized against inactivation in human serum, retaining > 90% activity after a 24-h incubation, whereas GLA showed < 10% activity after incubation in serum for the same time (Fig. 2C). Similarly, GLAv05 and GLAv09 retained > 90% activity after a 48-h incubation in lysosomal extract whereas GLA showed < 50% residual activity following the same challenge (Fig. 2D).

**Table 1:** Melting temperatures measured by differential scanning fluorimetry.

Enzyme	T_m_ (°C) pH 4.5	T_m_ (°C) pH 7.4
GLA	51.2	43.5
GLA + 50 µM DGJ	65.0	52.0
GLAv05	63.2	51.0
GLAv09	64.3	48.2

DGJ = 1-deoxygalactonojirimycin.

### Cellular uptake, stability, and activity of GLA variants

We next tested whether increased stability in vitro would translate to greater perdurance of activity in cells. We incubated Fabry fibroblasts with either GLA, GLAv05, or GLAv09 for 4 h, removed free enzyme and then assayed cell lysates for alpha galactosidase activity. Lysates from GLAv09-treated cells had 2-fold more activity than those treated with GLA, while lysates from GLAv05-treated cells had around 2-fold reduced activity (Figure S3). Given the higher in vitro stability of GLAv05 compared to GLA, this result suggested that the cell uptake of GLAv05 may be impaired as compared to GLA and GLAv09. We were unable to directly measure GLA protein uptake in cells using a commercial anti-GLA antibody because of markedly different affinity of the antibody to GLA and the two variants, GLAv05 and GLAv09 (Figure S6).

**Figure 3:**
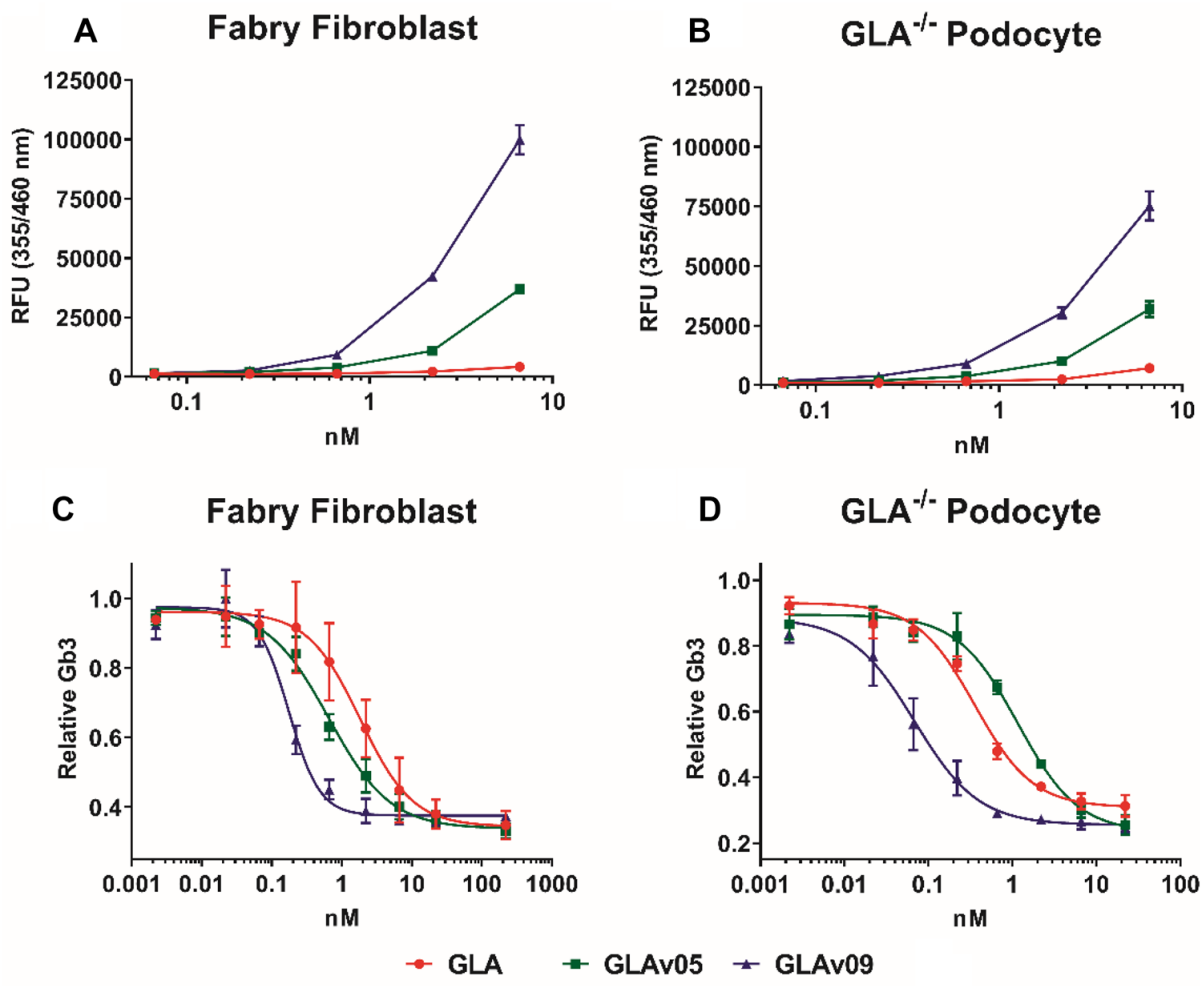
GLA activity and Gb3 degradation in cells following treatment with GLA variants. Cells were treated with GLA (red circles), GLAv05 (green squares), or GLAv09 (blue triangles), incubated for 7 days, then lysed for analysis. 4-MU-Gal hydrolysis activity of lysates (**A**,**B**) and LC-MS quantitation of Gb3 substrate (**C**,**D**) is shown for Fabry patient fibroblasts (**A**,**C**) and GLA^−/−^ podocytes (**B**,**D**).

Long-term cell uptake and stability of GLA was determined using cell lines with no intrinsic alpha galactosidase activity: either Fabry patient-derived fibroblasts or GLA^–/–^ podocytes^27^. Following incubation with GLA variants for 7 days, cell lysates were assayed for alpha galactosidase activity, and residual Gb3 content was quantified by mass-spectrometry^28^. Fabry fibroblasts lysates had 19-fold greater alpha galactosidase activity when treated with GLAv09 than with GLA control (Fig. 3A). Similarly, GLA^–/–^ podocytes treated with GLAv09 had 10-fold increased alpha galactosidase activity than GLA treated controls (Fig. 3B). Corresponding to the greater intracellular alpha galactosidase activity, GLAv09 effectively cleared Gb3 substrate from Fabry patient fibroblasts or GLA^–/–^ podocytes, with an approximately 10-fold reduction in IC50 as compared to GLA (Fig. 3C,D).

Exogenous GLA enters cells and is trafficked to the lysosome via the mannose-6-phosphate receptor (M6PR)-dependent pathway^29,30^. To determine whether GLAv09 enters Fabry fibroblasts by a M6PR-dependent pathway we tested whether Gb3 depletion activity could be blocked by competition with a large excess of mannose-6-phosphate (M6P). Consistent with M6PR-dependent trafficking to the lysosome, the decrease in Gb3 content of Fabry fibroblasts incubated with GLAv09 was completely blocked by the presence 10 mM M6P, but not by mannose (Figure S4).

### Mouse and non-human primate pharmacokinetics (PK) and pharmacodynamics (PD)

As the most rigorous test of GLA performance in the physiological context of a live animal, we determined the plasma PK properties of GLA, GLAv05 and GLAv09 in mice and cynomolgus monkeys (non-human primates, NHPs). We dosed animals at 1 mg/kg i.v. and followed enzyme activity in the plasma over time. This measure allowed us to follow the fate of active GLA enzyme but may underestimate the total amount (catalytically active and inactive) of circulating GLA protein.

**Figure 4:**
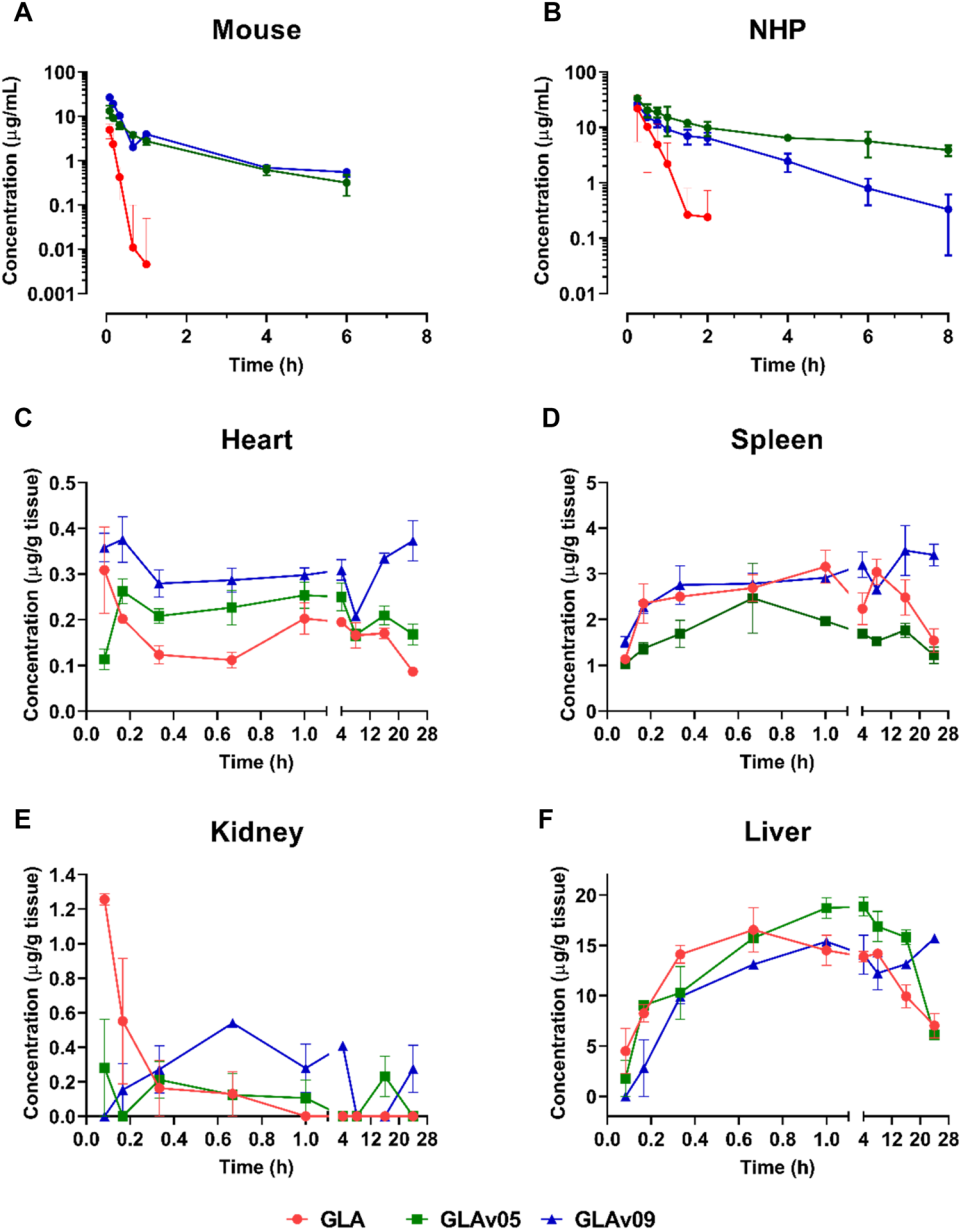
Pharmacokinetics and biodistribution of GLA variants. Healthy mice (**A**) and cynomolgus monkeys (**B**) were dosed at 1 mg/kg i.v. and plasma was analyzed for total active GLA. Hemizygous 2–3-month-old male Fabry  mice were treated with GLA and variants at 1 mg/kg i.v. At indicated timepoints, mice were sacrificed, perfused, and tissues were harvested, processed, and tested for alpha galactosidase activity to determine active enzyme concentration: heart (**C**), spleen (**D**), kidney (**E**) and liver (**F**).

As previously reported^31^, GLA activity in plasma decreased rapidly after administration and was undetectable after 1 h in mouse and after 2 h in NHP. In contrast, GLAv05 and GLAv09 exhibited much greater perdurance with measurable activity remaining 6 h after administration in mouse (Fig. 4A) and after 8 h in NHP (Fig. 4B). The half-lives in mouse of GLAv05 and GLAv09 were > 10-fold longer than for GLA. In NHP, GLAv05 and GLAv09 both showed terminal half-lives > 7-fold longer than GLA (Fig. 4A,B; Table 2).

**Table 2:** Mouse and Cynomolgus monkey plasma PK parameters represented by calculated half-life (t_1/2_), peak concentration in plasma (C_max_), area under the curve (AUC), clearance (CL), and volume of distribution (V_z_).

	Mouse			NHP		
	GLA	GLAv05	GLAv09	GLA	GLAv05	GLAv09
t_1/2_ (h)	0.15	1.58	1.70	0.19	5.20	1.32
C_max_ (μg/mL)	4.96	13.3	26.6	21.9	33.5	26.5
AUC_0-t_ (μg·h/mL)	1.26	12.37	18.25	10.2	108	35.2
CL (L/h/kg)	0.79	0.08	0.05	0.15	0.01	0.03
V_z_ (L/kg)	0.170	0.174	0.125	0.04	0.07	0.05

To determine whether GLAv05 and GLAv09 exhibit improved biodistribution in key tissues, we dosed Fabry mice at 1 mg/kg i.v. and sacrificed the animals at time points ranging from 5 min through 24 h to collect heart, kidney, spleen, and liver. As shown in Fig. 4C–F and Table 3, the GLA variants demonstrated improved tissue PK parameters as compared to GLA. The total amount of alpha galactosidase activity in the kidney for GLAv05 and GLAv09, as measured by the area under the curve (AUC), was 8–9-fold greater than for GLA, while heart activity for GLAv09 was 2-fold greater than for GLA. These results suggest that increased plasma half-life for a GLA enzyme translates to increased enzyme accumulation in the kidney and heart.

**Table 3:** Mouse tissue AUC_0-24_ (µg·h/g).

Tissue	GLA	GLAv05	GLAv09
Kidney	0.46	3.58	4.11
Heart	3.84	4.81	7.23
Liver	276	358	324
Spleen	59.2	38.9	75.7

**Figure 5:**
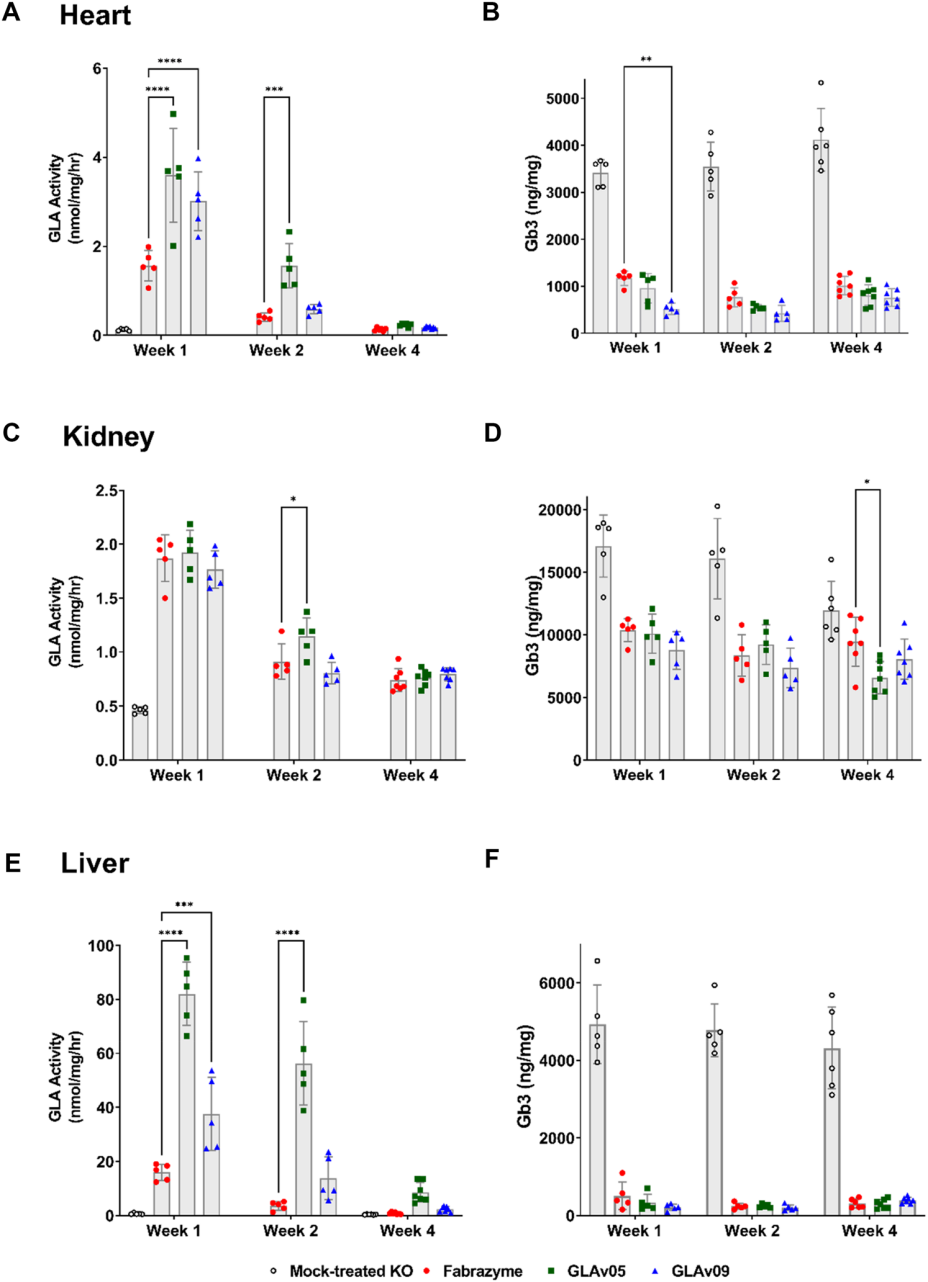
Pharmacodynamics (PD) alpha galactosidase activity and Gb3 accumulation after exposure to GLA variants. 5-month-old female GLA^−/−^ mice were dosed with GLA variants at 1 mg/kg i.v. At indicated timepoints, the mice were sacrificed, perfused, and tissues were harvested: liver (**A**,**B**), heart (**C**,**D**), and kidney (**E**,**F**). Tissues were processed and tested for alpha galactosidase activity (**A**,**C**,**E**) and Gb3 (**B**,**D**,**F**). Note that alpha galactosidase activity in liver is considerably higher than for other organs. *p < 0.05, **p < 0.01, ***p < 0.001, ****p < 0.0001—two way ANOVA with Dunnett multiple comparisons.

Finally, we sought to determine whether increased enzymatic activity in the kidney and heart of GLAv05 and GLAv09 leads to a reduction of Gb3 in these key affected organs compared to the standard of care ERT agalsidase-beta (Fabrazyme^®^). We intravenously dosed 5-month-old female mice with GLA variants at 1 mg/kg, then sacrificed the animals at 1, 2, or 4 weeks and harvested the heart, kidney, and liver for analysis of Gb3 content. GLAv05 and GLAv09 showed increased activity in the heart relative to GLA (agalsidase-beta, Fabrazyme^®^) (Fig. 5A) (and at levels similar to endogenous GLA in a healthy mouse^32^), which translated to enhanced Gb3 depletion for GLAv09, but not for GLAv05 (Fig. 5B). The superior efficacy for GLAv09 in the heart was sustained over four weeks, though the enzymatic activities for all treatment groups had reverted to near baseline by this time point. At 1- and 2-weeks post-treatment, GLAv09 depleted 84% and 88% of Gb3, respectively, compared to 65% and 77% for agalsidase-beta. Conversely, although GLAv05 showed increased activity in the heart at 1 and 2 weeks, this did not translate to an improvement in Gb3 depletion compared to agalsidase-beta.

All three GLA enzymes showed similar activity and Gb3 depletion profiles in the kidney, though we observed higher activity for GLAv05 at 2 weeks, which translated to statistically lower Gb3 levels after 4 weeks (Fig. 5C,D). In the liver, GLAv05 and GLAv09 showed 4- and 2-fold increased activity relative to agalsidase-beta at 1-week post-dose (Fig. 5E), but only the increased activity for GLAv09 correlated with a significant reduction in liver Gb3 (Fig. 5F). At 2 weeks following treatment, GLAv05 activity in liver was still significantly higher than the other groups, though Gb3 levels were similar across all groups. By week 4, alpha galactosidase activity was negligible for most mice in the agalsidase-beta and GLAv09 groups, and highest in the GLAv05 group. Together, the in vitro and in vivo pharmacology studies suggest favorable properties for GLAv05 and GLAv09 as compared to GLA and support their development as next-generation therapies for the treatment of Fabry disease.

### Directed evolution to reduce GLA immunogenicity

**Figure 6:**
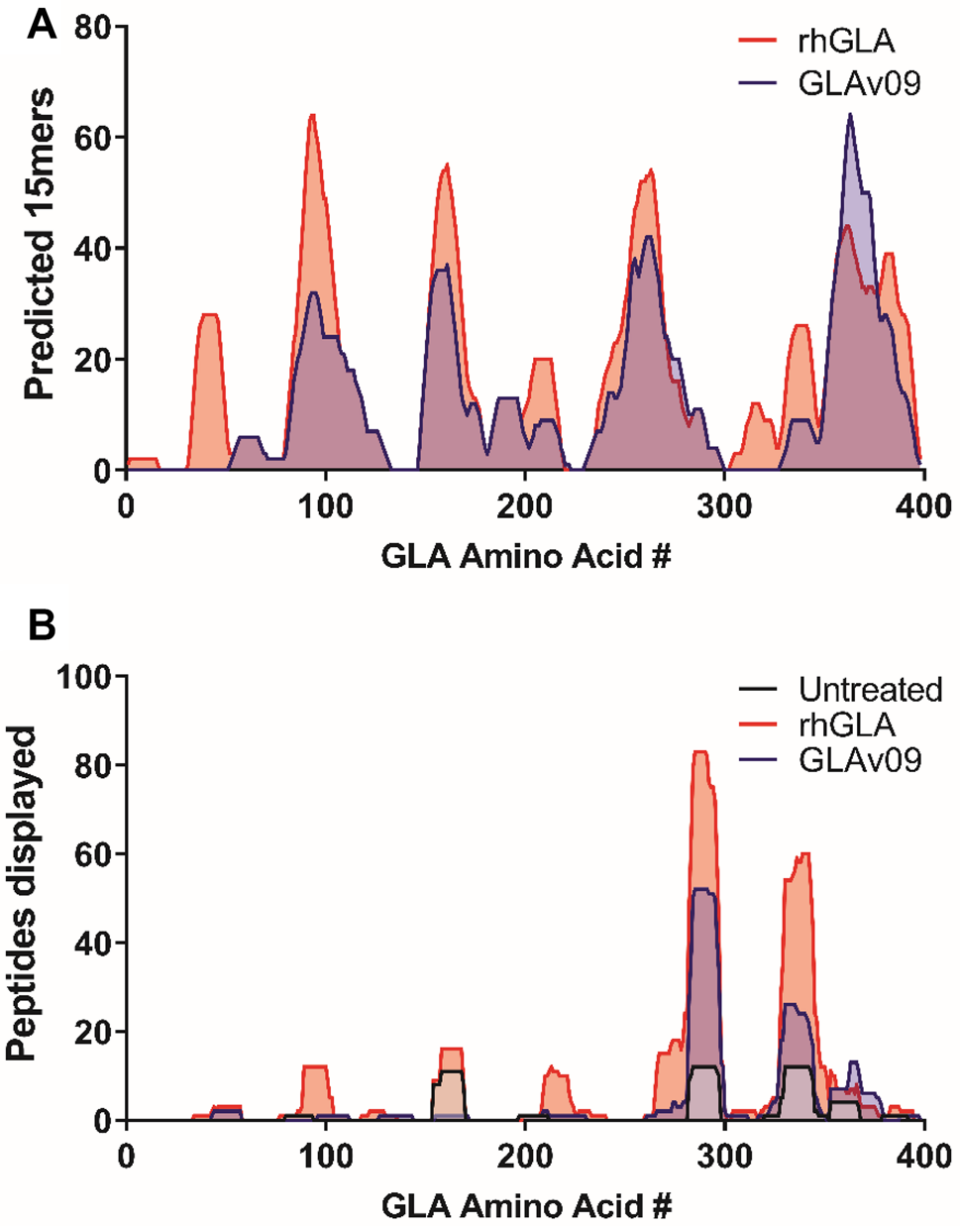
In silico and in vitro de-epitoping of GLA. (**A**) In silico assessment of 15-mer peptides of GLA (red) and GLAv09 (blue) using the IEDB software (https://www.iedb.org). GLA and GLAv09 sequences were parsed into all possible 15-mer analysis frames, with each frame overlapping the previous one by 14 amino acids. The 15-mer analysis frames were evaluated for immunogenic potential by scoring their 9-mer core regions for predicted binding to eight common MHC-II HLA-DR alleles (DRB1*0101, DRB1*0301, DRB1*0401, DRB1*0701, DRB1*0801, DRB1*1101, DRB1*1301, and DRB1*1501). (**B**) MHC-associated peptide proteomics of peptides displayed on dendriticcells from buffer (grey), GLA (red), and GLAv09 (green). Histograms indicate the number of displayed peptides comprising the indicated amino acid across the entirety of the GLA sequence.

Our strategy for diminishing the propensity of GLA to elicit an immune response in Fabry patients who have no endogenous GLA and thus lack immune tolerance for the protein was to eliminate GLA peptide sequences that can bind to MHC-II complexes. Accordingly, we used IEDB tools (https://www.iedb.org) to predict MHC-II binding of all overlapping 15-mer peptides derived from GLA^25,33^, then we incorporated mutations predicted to eliminate putative MHC-II binding peptides into the cycles of directed evolution. GLAv05 was specifically engineered to minimize the number of predicted MHC-II binding peptides, resulting in GLAv09, which has 17 mutations relative to GLA. Our in silico analysis predicted that GLAv09 would lead to presentation of fewer peptides by MHC-II as compared to GLA (Fig. 6A).

To empirically assess antigen processing, we tested variants in MHC-associated peptide proteomics (MAPPs) assays^34,35^. Briefly, monocyte-derived dendritic cells from healthy donors were incubated with GLA, GLAv09, or vehicle and MHC-II HLA-DR-bound peptides were characterized by mass spectrometry. Since the immune cells used in this assay are from healthy donors, it was important to show that there are very low background levels of endogenously derived GLA-specific peptides bound to MHC-II complexes. As shown in Fig. 6B, fewer HLA DR-bound peptides were derived from dendritic cells exposed to GLAv09 as compared to GLA.

### Assessment of neo-antigens in GLA variants

A possible unwanted consequence of the directed evolution strategy is that mutations may form neo-epitopes that generate an immune reaction in Fabry patients that express a full length GLA with low alpha galactosidase activity (cross-reactive immunogenic material positive or CRIM+). We used a T-cell proliferation assay that provides an assessment of the CD4+ T-cell response elicited by exposure to GLA, GLAv05, or GLAv09^36,37^.

**Figure 7:**
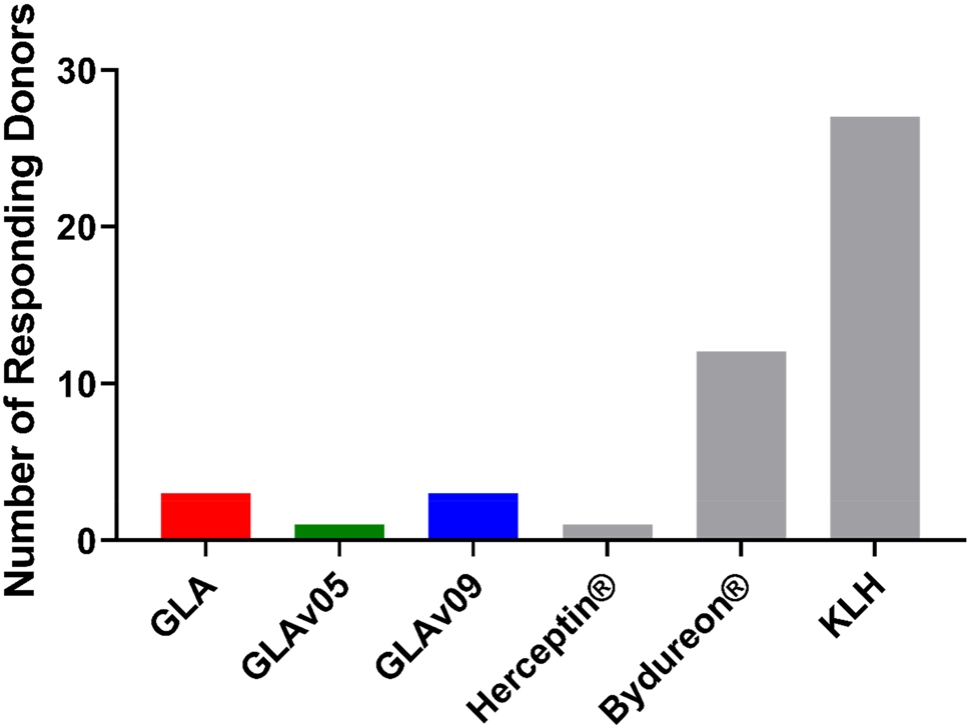
T cell response evoked by GLA variants. PBMCs from 30 healthy donors were treated with GLA, GLAv05, GLAv09, or controls in triplicate, and proliferation of T cells (in the presence of an inhibitor of CD8^+^ T cellswas measured by ^3^H-thymidine incorporation on days 5, 6, 7, and 8. Donors showing a response on any day were considered positive.

Peripheral blood mononuclear cells (PBMCs) derived from 30 healthy donors were treated with GLA, GLAv05, GLAv09, or controls, and T-cell proliferation was measured by [^3^H]-thymidine incorporation on days 5, 6, 7, and 8. GLA induced T-cell proliferation in 3/30 donors in GLA and GLAv09, where in GLAv05 induced T-cell proliferation in only 1/30 donors (Fig. 7). These response rates were significantly lower than those observed for the clinical benchmark control Bydureon (12/30) and the highly immunogenic neoantigen Keyhole limpet hemocyanin (KLH) positive control (27/30). Thus, the 11 mutations in GLAv05 and the 17 mutations in GLAv09 do not cause an increase in T-cell proliferation relative to the wild type GLA enzyme in healthy donor PBMCs.

## Discussion

We employed directed evolution to address the therapeutic shortcomings of GLA for the treatment of Fabry disease by engineering two variants, GLAv05 and GLAv09, for improved stability in serum and lysosomes and increased intracellular activity in target tissues. These variants also have a similar or lower predicted propensity to induce immune responses as compared to the wild-type enzyme^4,38–46^. Variants GLAv05 and GLAv09 demonstrate improved in vivo circulating half-life, tissue activity, and Gb3 clearance in the Fabry mouse model due to the translatability of in vitro directed evolution.

Various other approaches toward overcoming GLA limitations have been explored. Pegunigalsidase alfa, a PEGylated, cross-linked recombinant human GLA enzyme produced in plant cells^47^ is being evaluated in Phase 3 clinical trials. The chemical modifications of pegunigalsidase alfa confer stability in human plasma (30% activity after 1 h compared to complete inactivation of GLA) and a 9.7 h terminal half-life in GLA^−/−^ mice following a 1 mg/kg infusion^48^. The plasma stability of GLAv05 and GLAv09 compare favorably to pegunigalsidase alfa, as both retain > 80% activity after 24 h, though PEGylation appears to improve the serum half-life of pegunigalsidase alfa relative to our engineered variants. Future studies directly comparing these enzymes would be helpful toward understanding how these parameters may impact tissue distribution and efficacy.

Other approaches for improving the intrinsic limitations of GLA include rational mutagenesis of cysteine residues^49^, active site, dimer interface, and glycosylation sites^50^, deletion of C-terminal sequences^51^, rational modification of alternative enzymes^32^, the use of alternative expression hosts, such as moss^52^ and tobacco cells^48^, glycoengineering to improve affinity to the M6P receptor or increase M6P moieties on the enzyme^32,53–55^, and fusion proteins for improved tissue targeting^56,57^. While each of these approaches addresses aspects of intrinsic GLA shortcomings, our approach allows for the comprehensive optimization of multiple properties simultaneously via high-throughput screening of variant libraries under conditions that mimic the path the therapy is exposed to and the natural inactivation strategies it may encounter in the human body upon administration as ERT.

Given that endogenous GLA is synthesized in the endoplasmic reticulum and then translocated to the lysosome where it performs its function, it is not surprising that its extracellular half-life is limited. However, therapeutic GLA is produced via secretion from a cell line during biomanufacturing, purified, cold stored until administration, and infused, after which it is transported throughout the body and endocytosed by cells and trafficked to the lysosome. It is unlikely that GLA evolved naturally to withstand these steps, and the commercial drug production process provides a risk of causing a deterioration of ERT efficacy^13–15,58,59^. In our approach to generate GLA variants with better therapeutic properties, we focused on improving serum and lysosomal stability to increase both bioavailability and efficacy within the cell. Improving the stability of GLA at the physiological pH of plasma (pH 7.4) was deemed important to increase the bioavailability of active enzyme in circulation, while improving stability at acidic pH should increase the enzyme’s half-life in the lysosome. Through directed evolution, we improved the stability of the enzyme across the pH 4.2–7.4 range without affecting its kinetic properties. We avoided modifying known glycosylation sites to ensure that the mannose-6-phosphate uptake mechanism was retained.

In addition to introducing mutations that improve stability, we identified and incorporated mutations that reduced or eliminated in silico-predicted immunogenic epitopes^25,33^ and confirmed a reduction in putative epitopes by MHC-II presentation (MAPPs) assays. We further assessed GLAv05 and GLAv09 in T cell proliferation assays using PBMCs from healthy donors, where we observed responses in an equal (GLAv09) or fewer (GLAv05) number of donors as compared to GLA. Together, the MAPPs and T cell assay data suggest that GLAv05 and GLAv09 have similar or reduced preclinical immunogenicity risk profiles as compared to GLA.

The enhanced stability and increased cell uptake properties translated to improved PK and PD responses in animals given a single dose of GLAv05 or GLAv09. Interestingly, while the amounts of GLA, GLAv05, and GLAv09 accumulating in the liver over 24 h were similar (Fig. 4F; Table 3), after 1 week there was substantially more GLAv05 or GLAv09 in the liver as compared to GLA (Fig. 5E). We hypothesize that this may be due to the greatly enhanced stability of the GLAv05 and GLAv09, leading to slower degradation and increased retained activity over time as compared to GLA. This increased accumulation and concomitant reduction in Gb3 (Fig. 5E,F) was also observed in heart (Fig. 5A,B), a key impacted organ in Fabry patients^3,39,43^. Conversely, it is notable that despite an 8–9-fold improved exposure in the kidney for GLAv05 and GLAv09, the two variants do not show significant Gb3 reduction over GLA in this organ. In the future it will be important to evaluate these enzymes in long-term, multiple dose studies in Fabry disease animal models to determine whether the short-term observations in tissue activity and Gb3 depletion translate into phenotypic benefits.

Regarding the therapeutic potential of engineered proteins as ERTs, there are prior examples of mutant proteins used clinically as replacement therapies. Most notably, the Factor IX Padua variant^60^, a naturally occurring Factor IX variant with increased activity has been shown to improve the efficacy of hemophilia B gene therapy by up to 10-fold in preclinical studies^61^ and has shown promising results in clinical trials^62^. While Factor IX Padua has only a single mutation relative to wildtype Factor IX and its discovery was serendipitous^60^, here we have shown that directed evolution can be used to accelerate the identification of protein variants with improved stability and potential as effective therapeutics. Because the changes in GLAv05 and GLAv09 are in their primary sequence, their therapeutic effect may be exerted via administration as recombinant biologic, gene, mRNA, or cell therapy. A more efficacious GLA enzyme with improved pharmacokinetics in body fluids and tissues could, in principle, reduce the dosing frequency or increase the efficacy for an enzyme replacement therapy. Alternatively, as a gene therapy, an improved enzyme potentially could be delivered with a lower dose of AAV.

In addition to providing potential new treatment candidates, evolved enzymes are also interesting tools that may help advance our understanding of α-galactosidase, Fabry disease, and lysosomal biology, such as the molecular aspects of intracellular trafficking, interactions with cellular uptake or chaperone proteins such as saposins, and the immune response to enzyme replacement therapies.

In summary, using directed evolution, and after screening ~ 12,000 GLA variants, we identified GLAv05 and GLAv09 as highly efficacious GLA variants with potentially reduced immunogenicity risk profiles. Compared to GLA, GLAv05 and GLAv09 displayed improved in vitro biophysical properties and intracellular function. Furthermore, GLAv05 and GLAv09 displayed improved PK, PD, and efficacy in the Fabry mouse model. Our results suggest that directed evolution technologies provide a way to discover new treatments for Fabry disease that overcome the shortcomings of ERTs that are based on the natural human enzyme sequence. This approach is likely applicable to lysosomal storage disorder treatments in general.

## Materials and methods

### Construction of expression vectors

The human GLA gene was codon optimized for expression in *Saccharomyces cerevisiae*. A chimeric GLA expression construct encoding a 19-amino acid *S. cerevisiae* MF-alpha signal sequence fused to the mature form of the codon-optimized GLA was generated in the yeast expression vector pYT-72Bgl^63^ and was utilized in rounds 1 through 7. A chimeric GLA expression construct encoding a synthetic mouse Ig signal sequence fused to a synthetic gene coding for the mature human GLA coding sequence (either *S. cerevisiae* or human codon optimized) was generated in pcDNA 3.1(+) (Invitrogen) for expression of recombinant GLA variants in HEK293T cells. For round 8 of directed evolution, transcriptionally active PCR products for the GLA variants encompassing promoter, GLA gene, and terminator were used for high-throughput transient transfections.

### Library design and generation

Libraries of variants with single amino acid mutations as well as combinatorial libraries recombining multiple mutations, were generated by standard mutagenesis methods^64^ using PCR and degenerate oligos (i.e. NNK, NNN, NNS) to incorporate mutations at the targeted position.

### HTP expression of GLA variants in yeast

Yeast (INVSc1) cells (Catalog #C81000, ThermoFisher Scientific), transformed with vectors expressing GLA variants using the lithium acetate procedure^65^, were plated on SD-Ura medium (2 g/L SD Media—Ura, 6.8 g/L yeast nitrogen base without amino acids [Sigma Aldrich]), 3.06 g/L sodium dihydrogen phosphate, 0.804 g/L disodium hydrogen phosphate (pH 6.0) 6% glucose. After 72 h incubation at 30 °C, colonies were picked into the wells of Axygen^®^ 1.1 mL 96-well deep well plates containing 200 µL/well SD-Ura. The cells were grown for 20–24 h in a Kuhner shaker (250 rpm, 30 °C, and 85% relative humidity). Overnight culture samples (20 µL) were transferred into Coming Costar^®^ 96-well deep plates filled with 380 µL of SD-Ura broth supplemented with 2% glucose. The plates were incubated for 66–84 h in a Kuhner shaker (250 rpm, 30 °C, and 85% relative humidity). The cells were then pelleted (4000 rpm × 20 min), and the supernatants isolated for immediate analysis, or stored at 4 °C prior to analysis.

### HTP expression of GLA variants in HEK293T cells

HEK293T cells (ATCC CRL-1573) were plated in 200 μL of DMEM with 10% FBS and grown with 5% CO_2_ at 37 °C in a 96-well Corning plate at 75% confluency 24 h prior to transfection. Plasmids containing GLA variant genes were purified using the QIAGEN Plasmid Plus 96 miniprep kit. HEK293T cells were transfected with plasmid for each variant, into individual wells according to the standard transfection protocol for Lipofectamine 3000 (ThermoFisher). Transfected cells were incubated for 72–96 h and the conditioned media was removed and assayed immediately as described below.

### In silico T-cell epitope prediction

Putative T-cell epitopes in GLA, GLAv05 and GLAv09were identified using the Immune Epitope Database (iedb.org) tools^25^. The GLA, GLAv05 and GLAv09 sequences were parsed into all possible 15-mer segments, with each segment overlapping the previous one by 14 amino acids. Each 15-mer was evaluated for immunogenic potential by scoring their 9-mer core regions for predicted binding to eight common MHC-II HLA-DR alleles (DRB1*0101, DRB1*0301, DRB1*0401, DRB1*0701, DRB1*0801, DRB1*1101, DRB1*1301, and DRB1*1501).

### MAPPS assay

MAPPs (MHC-associated peptide proteomics) assays were performed at Lonza Ltd. following their established protocols^34,66^. In brief, monocytes were isolated from frozen peripheral blood mononuclear cells (PBMCs) samples by positive magnetic bead selection and differentiated to dendritic cells (DCs). DCs were then loaded with the test protein and matured with LPS for 24 h. After maturation the DCs were lysed and the membrane fraction containing the HLA:peptide complexes was solubilized and incubated with Protein A sepharose beads coated with monoclonal antibody to MHC II-DR. Following elution by 0.1% TFA, the peptides were desalted by passage through a 10 kDa MWCO spin column and stored at − 80 °C for MS analysis. All blood cells and monocytes were collected by Lonza (license number 12590) in accordance with the Human Tissue Authority, the regulatory body for the Human Tissue Act 2004. This act regulates the removal, storage, use and disposal of human tissue within the UK. Accordingly, all activities that fall under this act were performed to HTA standards, with donors consent.

### T-cell proliferation assay

The T cell proliferation assay was performed at Abzena Ltd. Abzena (Cambridge) Ltd is licensed (number 12627) by the Human Tissue Authority, the regulatory body for the Human Tissue Act 2004. This act regulates the removal, storage, use and disposal of human tissue within the UK. Accordingly, all activities that fall under this act were performed to HTA standards, with donors consent. As follows: PBMCs were isolated from 30 healthy human donors selected to cover all major HLA-DR and HLA-DQ allotypes. PBMCs were depleted of CD8+ T cells using CD8+ RosetteSepTM (StemCell Technologies Inc, London, UK). Three samples, (GLA, GLAv05 and GLAv09) and three controls (KLH: positive control, Bydureon^®^ (AstraZeneca, UK): a clinical benchmark control, and Herceptin^®^ (Roche, Switzerland): a low immunogenicity control) were used to treat each donor sample. Cultures were incubated for a total of 8 days at 37 °C with 5% CO_2_. On days 5,6,7, and 8, aliquots were transferred to a round bottomed 96 well plate and cultures were pulsed with [3H]-Thymidine (Perkin Elmer^®^, Beaconsfield, UK) and incubated for another 18 h before harvesting. [3H] cpm for each well were determined by Meltilex™ (Perkin Elmer^®^, Beaconsfield, UK) scintillation counting on a 1450 Microbeta Wallac Trilux Liquid Scintillation Counter (Perkin Elmer^®^, Beaconsfield, UK) in paralux, low background counting. For proliferation (n = 3 per time point), positive responses were defined by statistical and empirical thresholds as follows: (1) significance (p < 0.05) of the response by comparing cpm or spw of test wells against control wells using unpaired two sample Student’s t-test. (2) SI ≥ 1.90, where SI = mean of test wells (cpm or spw)/baseline (cpm or spw). Donors that were positive on at least one time point during the time course assay were deemed positive donors. P values were calculated using repeated measures one-way ANOVA with Friedman’s multiple comparison post-tests in Prism 8 (GraphPad, La Jolla, USA).

### GLA expression and purification

Expi293 cells (Thermo Fisher) were seeded in shake flasks and grown in serum-free chemically defined medium. Expi293 cells in suspension (1 L) were transfected with expression constructs for GLA variants and cells were harvested at day 5 post-transfection. Affinity purification of GLA variant purification was performed based on protocols described by Yasuda et al.^67^. In brief, culture supernatants clarified by filtration through 0.2 µm membranes were applied to concanavalin A–Sepharose pre-equilibrated with 0.1 M sodium acetate buffer (pH 6.0) containing 0.1 M NaCl, 1 mM MgCl_2_, 1 mM CaCl_2_, and 1 mM MnCl_2_. GLA bound to concanavalin A were eluted with 0.1 M sodium acetate buffer (pH 6.0) containing 0.1 M NaCl, 1 mM MgCl_2_, 1 mM CaCl_2_, 1 mM MnCl_2_, and 0.9 M methyl-d-mannopyranoside, and 0.9 M methyl-d-glucopyranoside. Fractions of the homodimer were pooled and concentrated. For polishing by size-exclusion chromatography (SEC), protein was concentrated and loaded onto a Superdex 200 PG 26/60 column. The protein was eluted with 2.4 mM Na_2_HPO_4_, 17.6 mM NaH_2_PO_4_ (pH 6.0) 150 mM NaCl, 0.02% Tween-20, at a flow rate of 2.5 mL/min. Purified protein (> 95% pure by SEC) was stored at − 80 °C until use.

### GLA activity assay

GLA, GLAv05 and GLAv09 (50 µL at 100 nM) were transferred in triplicate into a black 96-well plate containing 50 µL of 1.5 mM 4-methylumbelliferyl-d-galactopyranoside (4-MU-Gal) in McIlvaine Buffer (pH 4.4). Plates sealed with plateLoc microplate heat seals were incubated for 2–4 h with agitation (400 rpm) at 37 °C. Reactions were quenched by addition of 100 µL of 0.5 M Na_2_CO_3_ (pH 10.5), and fluorescence (355/460 ex/em) was quantified using a SpectroMax Plus 384 (Molecular Devices) plate reader. Data were analyzed using SoftMaxPro and Prism software.

Reactions containing 15 nM enzyme were initiated simultaneously upon substrate addition and quenched at 5, 10, or 15 min. Michaelis–Menten kinetic parameters V_max_, k_cat_, and K_M_ were determined from enzyme activity at eight MU-Gal concentrations, ranging from 0.1 to 7.5 mM (n = 3 for each time point and concentration). The initial rate of enzyme activity as a function of substrate concentration was fitted to kinetic parameters using GraphPad Prism.

### Human serum, temperature, lysosomal and pH stability assays

For GLA stability assessment in human serum, 90 µL of human serum (Innovative Research Pooled Human Serum) was added to enzyme at a concentration of 1 µM in 10 µL PBS (pH 6.2)]. 96-well plates were sealed with plateLoc microplate heat seals and incubated for either 2, 4, 24 or 48 h at 37 °C with agitation at 300 rpm. At the conclusion of each stability challenge, samples were diluted 1:1 into alpha galactosidase activity buffer to assess residual active enzyme. Data was normalized to variant activity in the absence of serum.

For assessment of temperature stability, GLA, GLAv05 and GLAv09 were diluted to 5 µg/mL in PBS (pH 6.2), aliquoted into a 96-well PCR plate and heated at constant temperatures of 30.1, 31.8, 33.7, 36, 38.6, 41.2, 43.9, 46.3, 48.2, or 49.6 °C in a thermocycler for 1 h. Activities were normalized to the 30 °C-incubated sample to determine percent residual activity.

For T_M_ measurement by differential scanning fluorometry, GLA enzymes diluted to 1 mg/mL in PBS (pH 6.2) were mixed with 40 µL of pH 4.4 or pH 7.4. McIlvaine Buffer containing 1× or 5× SYPRO Orange was mixed with 10 µL of each enzyme solution (n = 3) ± 1-deoxygalactonojirimycin (DGJ) in separate wells of 96-well skirted Biorad plates. The plates were sealed with optically clear film and run on a CFX connect real-time PCR system using the manufacturer’s recommended method from 25 to 95 °C. Data were analyzed by BioRad Software.

For GLA stability assessment in human liver lysosomes, GLA, GLAv05 and GLAv09 were diluted to 5 µg/mL in 1X Catabolic buffer with human liver lysosomal extract (Xenotech), 96-well plates were sealed with plateLoc microplate heat seals and incubated for either 2, 24 or 48 h at 37 °C. At the conclusion of each stability challenge, samples were diluted 1:1 into alpha galactosidase activity buffer to assess residual active enzyme. Data was normalized to variant activity in the absence of challenge.

For assessment of pH stability, GLA, GLAv05 and GLAv09 were diluted to 50 µg/mL in PBS pH 6.2. 45 µL of McIlvaine buffer (prepared at pH 2.2, 3.0, 4.0, 4.6, 5.0, 5.6, 6.0, 6.6, 7.0, 7.4 or 8.0) and 5 µL of each enzyme solution were incubated at 37 °C, 400 rpm for 0, 2, 4, 6, and 24 h, samples were then diluted 1:1 into alpha galactosidase activity assays to assess residual active enzyme. Activities were normalized to the activity across the pH profile and plotted.

Experiments were done in triplicate for pH, lysosomal, serum stability and melting temperature experiments; data for temperature stability are from a single trial.

### GLA uptake, activity, and gb3 quantitation in cultured cells

GLA^–/–^ podocytes^27^ or Fabry patient-derived fibroblasts (NIGMS Human Genetic Cell Repository at the Coriell Institute for Medical Research, cell line GM02775) were plated in 200 µL complete growth medium (1:1 DMEM:F-12 medium with 10% heat-inactivated FBS, 1% (vol/vol) insulin-transferrin-selenium (Gibco) (1 mg/mL, 0.55 mg/mL, and 0.67 μg/mL, respectively), and 1% (vol/vol) penicillin/streptomycin (10,000 U/m) or MEM with 1% NEAA and 15% heat-inactivated FBS, respectively) and allowed to adhere to the plastic substrate. Once cells reached 90% confluency, culture medium was removed, and cells were incubated with GLA, GLAv05 and GLAv09 (at each concentration indicated from 0 to 200 nM) in complete medium. At the conclusion of treatment, media and residual GLA were removed, cells were washed 2× with 150 µL of PBS, and 200 µL of standard complete growth medium was added to each well. Plates were returned to the incubator for the remaining treatment time. Cells were harvested, lysed, and alpha galactosidase activity in the cell lysate was measured as described above. For measuring cellular Gb3 content, following treatment and incubation, the medium was removed, and cells were washed 2 × with PBS. The plates were frozen at − 20 °C. Gb3 was extracted with 200 µL methanol that included a 1 ng/µL C17 Gb3 standard (Matreya LLC), for 30 min at room temperature with gentle agitation. The entire 200 µL sample was transferred to a Costar round plate, sealed, and Gb3 analyzed on a 4000 Q-trap MS, as described previously^68^.

### In vivo studies

All procedures were conducted in compliance with the Animal Welfare Act Regulations (9 CFR 3) and were reviewed and approved by the appropriate Institutional Animal Care and Use Committees at the Institute of Metabolic Disease, Baylor Research Institute and Charles River Laboratories. The experiments were conducted in accordance with the ARRIVE guidelines. No significant clinical signs, moribundity, or mortality occurred throughout the studies. All animals were acclimated for at least 3 days prior to study start.

### Mouse PK

Healthy male C57Bl/6 mice (sourced from Charles River; aged 7–8 weeks, 25–30 g) were randomly distributed into groups for PK studies (n = 12). All animals received a single 1 mg/kg i.v. dose of either GLA or GLA variant, dosed based upon body weight, via the tail vein. Approximately 0.15 mL of blood was collected into heparinized capillary tubes (Fisher Scientific) at 5, 10, 20, 30, 40, 60, 240, and 360 min post dosing via tail bleed, alternating between two groups of 6 mice for each time point, and processed immediately for plasma according to manufacturer’s instructions. Plasma was transferred to a 96-well deep well plate and stored at − 80 °C for later analysis.

Hemizygous male Fabry mice, aged 2–3 months, (Stock #3535, Jackson Laboratories)^69^ were randomly distributed into groups for PK studies (n = 90). Following dosing, mice (n = 3 per time point) were anesthetized with isoflurane (deep anesthesia confirmed by a paw pinch) to perform a cardiac puncture for blood collection at 5, 10, 20, 40 min, and 1, 4, 8, 16, and 24 h post dose, perfused, then heart, spleen, kidney, and liver tissues were flash frozen and stored at − 80 °C until analysis of alpha galactosidase activity to determine active enzyme concentration in tissue.

### NHP PK

Healthy male protein-naïve cynomolgus monkeys, 2–3 kg body weight, were randomly distributed into groups for PK studies (n = 4). Animals received a single 1 mg/kg i.v. administration (tail or saphenous vein) of either GLA or GLA variant via use of a temporary placed catheter (24G angiocath). Blood (approximately 0.5 mL) was collected into K_2_-EDTA separator tubes (BD Biosciences) at pre-dose, 15, 30, 45, 60, 90 min, and 2, 4, 6, 8, 12, 24, and 48 h post-dosing, and stored on wet ice. Samples were processed to plasma following manufacturer’s instructions within 30 min of sample collection. Plasma was transferred to a 96-well deep well plate and stored at − 80 °C until analysis.

### Analysis of GLA enzymatic activity in plasma

Standard curves for each GLA variant were prepared in the appropriate animal plasma in a range from 0.819 to 200 ng/mL, including a negative control sample. For analysis, plasma samples were diluted 1:200 in McIlvaine buffer, pH 4.4. 50 μL of diluted samples were then manually pipetted and mixed 1:1 in a black 96-well plate with 1 μM MU-Gal in McIlvaine buffer, pH 4.4, to initiate the reaction. Following a 1-h incubation at 37 °C with agitation at 300 rpm, the reactions were quenched with 100 µL 0.5 M Na_2_CO_3_, pH 10.5. Fluorescent methylumbelliferol released by GLA was quantified spectrophotometrically (355/460 ex/em) using a SpectroMax Plus 384 (Molecular Devices) plate reader. Data were analyzed using SoftMaxPro and Prism.

### Tissue biodistribution and Gb3 depletion in fabry mouse

50 female Fabry (GLA^−/−^) mice (Stock #3535, Jackson Laboratories)^69^, aged 5 months, were used for these experiments (n = 5 per group). All animals received a single i.v. tail-vein administration of either buffer (mock-treated control), agalsidase-β, or GLA variant at a 1 mg/kg dose. Mice were anesthetized with isoflurane (deep anesthesia confirmed by a paw pinch) to perform a cardiac puncture for tissue perfusion at either 1, 2, or 4 weeks post i.v. administration. Liver, kidney and heart were harvested, frozen in dry ice, and stored at − 80 °C until analysis of GLA enzymatic activity and Gb3 levels in tissue.

### Analysis of GLA enzymatic activity in harvested tissue (Fabry mouse)

Mouse tissues were homogenized as described^70^. Tissue lysates were sonicated, centrifuged at 14,000 rpm for 15 min at 4 °C, and the supernatants used for enzyme assay. Alpha galactosidase activity was measured by the standard fluorometric assay^24^, using 5 mM 4-MU-Gal in McIlvaine buffer (pH 4.4), in the presence of 0.1 M *N*-acetylgalactosamine (a specific inhibitor of α-galactosidase B). Concentration of total protein was measured using the Pierce BCA protein assay kit (Thermo Fisher). Active GLA concentration for each variant was determined by its corresponding standard curve spiked in blank matrix and was expressed as ug/g tissue. Data were analyzed in GraphPad Prism using a two-way ANOVA with Tukey’s multiple comparisons between the mock-treated control group and GLAv05 and GLAv09 (Week 1 only), or between the agalsidase beta-treated group and GLAv05 and GLAv09.

### Analysis of Gb3 levels in harvested tissue (Fabry mouse)

Mouse tissues were homogenized and Gb3 content measured as previously described^70^. The lysates, normalized to 200 μg of total protein, were subjected to glycosphingolipids extraction, saponification, and subsequent analysis of Gb3 by mass spectrometry. Data were analyzed in GraphPad Prism using a two-way ANOVA with Tukey’s multiple comparisons between the mock-treated control group and GLAv05 and GLAv09, or between the agalsidase beta-treated group and GLAv05 and GLAv09.

## Supplementary Information

Supplementary Information.

## Data Availability

The data presented in the current study are available from the corresponding author on reasonable request.
