# Supplementary material for: Optimizing human α-galactosidase for treatment of Fabry disease
Source: Sci Rep. 2023 Mar 23;13:4748. doi: 10.1038/s41598-023-31777-4 (PMC10036536; doi:10.1038/s41598-023-31777-4)
Supplement: Supplementary file 1 — Supplementary Information. [file 41598_2023_31777_MOESM1_ESM.docx]

**Supplemental Section**

Supplemental Tables

**Table S1:** Michaelis-Menten kinetics of rhGLA and variants.

|  | rhGLA | GLAv05 | GLAv09 | Fabrazyme[1] |
| --- | --- | --- | --- | --- |
| Vmax (mmol/hr/mg) | 3.184 | 4.132 | 4.268 | 4.77 |
| k_cat_ (s^-1^) | 86.26 | 111.95 | 115.63 | n/a |
| K_M_ (mM) | 0.6171 | 0.7222 | 0.8947 | 2.0 |
| R^2^ | 0.955 | 0.956 | 0.958 | n/a |

**Supplemental Figures**

Figure S1


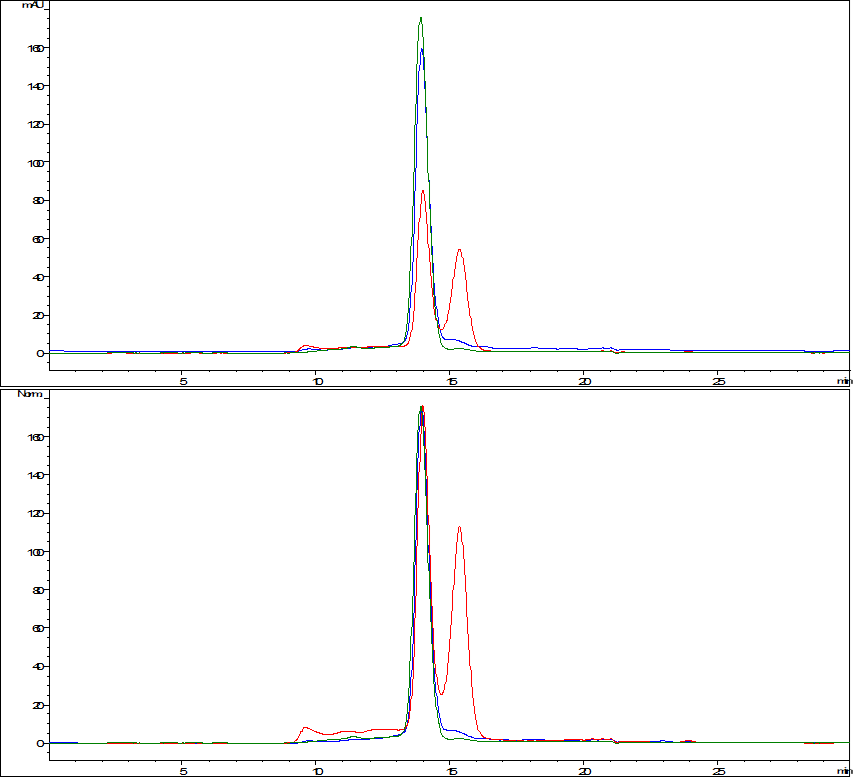


Red: rhGLA

Green: GLAv09

Blue: GLAv05

**Figure S1**. Analytical SEC traces of rhGLA, GLAv05, and GLAv09 following purification by concanavalin A chromatography.

Figure S2

**Figure S2.** Michaelis-Menten plot for GLA variants. Reactions containing 15 nM enzyme were initiated by addition of 4-MU-Gal substrate at indicated concentrations and quenched at 5, 10, or 15 minutes (n=3 for each time point and concentration).

Figure S3

**Figure S3.** Uptake of GLA variants into Fabry fibroblasts as measured by activity. Cells were treated with 10 ug/mL enzyme for 4 h, lysed, and the GLA activity was determined using 4-MU-Gal.

Figure S4


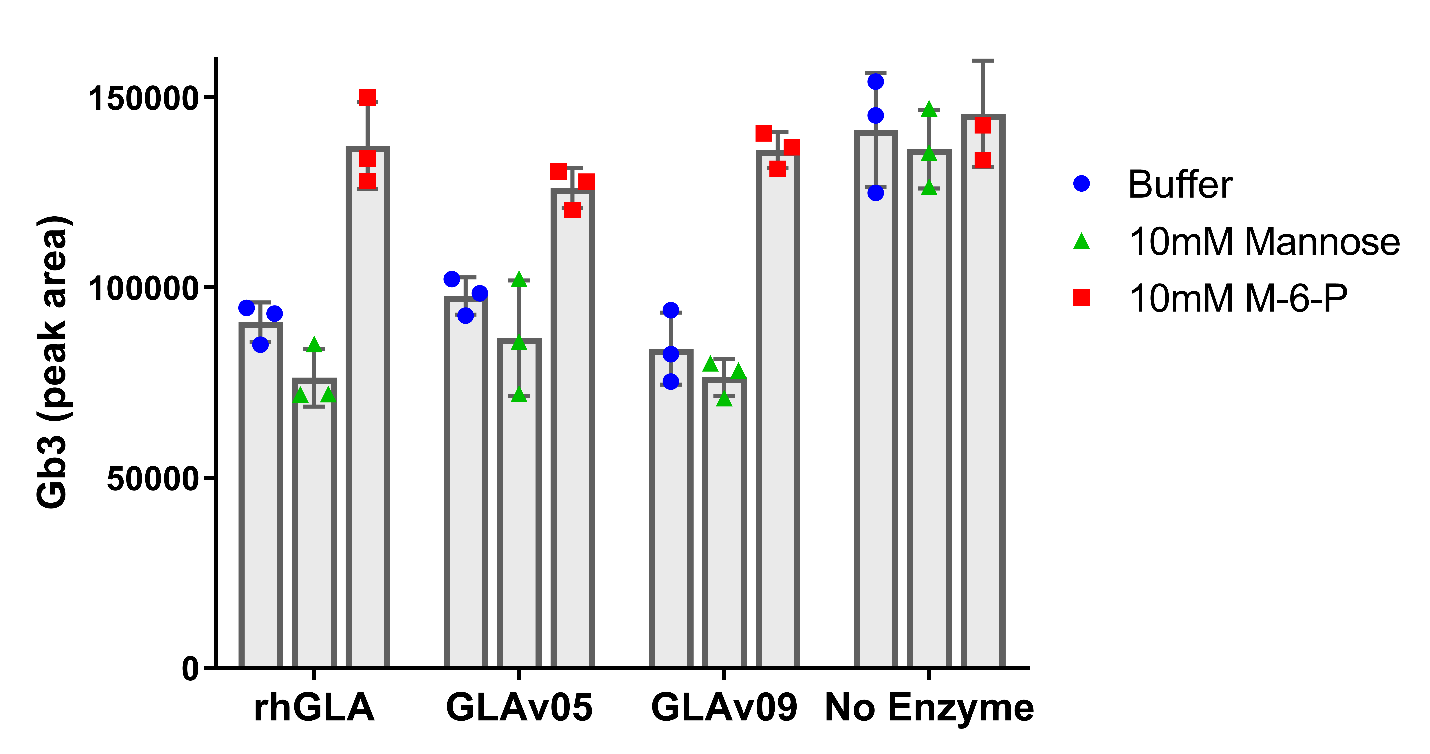


**Figure S4.** Inhibition of GLA uptake into Fabry fibroblasts after incubation of enzymes with mannose or mannose-6-phosphate. Cells were treated with 10 ug/mL enzyme in the presence or absence of 10 mM mannose or mannose-6-phosphate for 1 h, lysed, and Gb3 content was measured and compared to untreated cells.

Figure S5

**Figure S5.** Stability of GLA variants raw data. The activity of each variant (GLA (red circles), GLAv05 (green squares), and GLAv09 (blue triangles) was measured in a 4-MU-Gal hydrolysis assay following the indicated challenge conditions (three technical replicates per sample, except for heat challenge which shows results of a single test at each data point). **A.** Enzymes were pre-incubated at the indicated pH for 24h (n=3). **B.** Enzymes were pre-incubated at the indicated temperature for 1h (n=1). **C.** Enzymes were incubated in human serum at 37°C and assayed at indicated timepoints (n=3). **D.** Enzymes were incubated in human liver lysosome extracts at 37°C and assayed at indicated timepoints (n=3).

Figure S6

**Figure S6.** Binding affinity of GLA, GLAv05 and GLA v09 measured by ELISA. GLA and variants were diluted to the indicated concentrations and GLA quantification by ELISA was performed using the protocol of the Human Alpha-Galactosidase colorimetric sandwich ELISA kit from RayBiotech (part number ELH-AGLA-2).

**References**

1. Lee, K., et al., *A biochemical and pharmacological comparison of enzyme replacement therapies for the glycolipid storage disorder Fabry disease.* Glycobiology, 2003. **13**(4): p. 305-13.
